# Incidental Primary Hepatic Tuberculosis Mimicking Malignancy in a Patient With Hepatolithiasis: A Case Report of a Rare Surgical Condition

**DOI:** 10.7759/cureus.90000

**Published:** 2025-08-13

**Authors:** Lakshay Singla, Sharvari Pujari, Ramkrishna Y Prabhu, Chetan Kantharia

**Affiliations:** 1 Department of Surgical Gastroenterology, Seth Gordhandas Sunderdas (GS) Medical College and King Edward Memorial (KEM) Hospital, Mumbai, IND; 2 Department of Gastrointestinal Surgery, Seth Gordhandas Sunderdas (GS) Medical College and King Edward Memorial (KEM) Hospital, Mumbai, IND

**Keywords:** acute cholangitis, granulomatous inflammation, hepatolithiasis, histopathology, primary hepatic tuberculosis

## Abstract

Primary hepatic tuberculosis (TB) is an uncommon and diagnostically challenging condition, particularly in the absence of miliary or pulmonary disease. It can closely mimic hepatobiliary malignancies both clinically and radiologically, often leading to misdiagnosis and unnecessary surgical interventions. We report a case of a 45-year-old man who presented with right upper quadrant abdominal pain, fever, and jaundice. His medical history included open cholecystectomy with resection and anastomosis of the transverse colon, and endoscopic retrograde cholangiopancreatography for a choledochocolic fistula three years prior. Imaging revealed hepatolithiasis with left hepatic duct stricture and lobe atrophy, raising suspicion of malignancy. The patient underwent left lateral segmentectomy with ileal resection. Histopathological examination revealed nonnecrotizing granulomatous inflammation consistent with hepatic TB. Acid-fast bacilli staining was negative, and TB polymerase chain reaction could not be performed. The patient was started on antitubercular therapy and responded well. Given its rarity and nonspecific presentation, primary hepatic TB should be considered in the differential diagnosis of hepatic lesions in endemic regions. A high index of suspicion and the use of preoperative biopsy in equivocal cases can help avoid unnecessary surgical intervention and facilitate appropriate medical treatment.

## Introduction

Primary hepatic tuberculosis (TB) is a rare condition. TB can affect the liver either as part of miliary TB or as primary hepatic TB [1]. It can be classified into three main types: 1) miliary TB of the liver, which is most commonly associated with disseminated disease, 2) nodular (focal) hepatic TB, which may mimic tumors, and 3) tuberculous cholangitis, which is the least common and involves the bile ducts. Even in endemic regions, it remains extremely uncommon. It often mimics malignancy in terms of both clinical presentation and imaging findings [2]. Patients typically present with high-grade fever, abdominal pain, weight loss, and hepatomegaly [3]. Jaundice is rare in hepatic TB and often complicates the diagnostic process. Atypically, it can present as an isolated liver mass mimicking malignancy or abscess [4]. Due to the lack of specific clinical features and characteristic radiological findings, diagnosing this condition can be challenging. A definitive diagnosis is usually established through histopathology, which commonly reveals caseating epithelioid granulomas or acid-fast bacilli. We report a case diagnosed as primary hepatic TB through histopathology following hepatic resection for hepatolithiasis complicated by cholangitis.

## Case presentation

A 45-year-old man presented with complaints of dull, intermittent right upper quadrant abdominal pain for 15 days, accompanied by fever with chills, rigors, and progressive yellowish discoloration of the eyes, skin, urine, and clay-colored stools. There was no associated vomiting or significant respiratory symptoms. The patient exhibited no gastrointestinal symptoms such as chronic diarrhea and weight loss suggestive of intestinal TB. The patient had a normal nutritional status, with no signs of cachexia or lymphadenopathy, suggestive of chronic immunodeficiency.

The patient did not have any history of alcohol use or hemolytic disease. Along with this, there was no family history of liver disease and no known exposure to hepatotoxic drugs or industrial toxins. Given the unusual presentation of primary hepatic TB, the patient was evaluated for possible underlying immunodeficiency. As part of the workup, HIV testing was performed and returned negative. There was no history of immunosuppressive medication use, malignancy, or chronic comorbidities such as diabetes mellitus.

Based on the available clinical records and operative details, the patient was previously diagnosed with a choledochocolic fistula. The patient had a prior surgical history of open cholecystectomy with resection and anastomosis of the transverse colon for a choledochocolic fistula three years earlier, during which endoscopic retrograde cholangiopancreatography and biliary stenting were also performed. The details were obtained from the discharge summary, which was reviewed and included in the patient's records at our center. He was lost to follow-up postoperatively and remained asymptomatic until the current episode.

On clinical examination, the patient was afebrile with stable vital signs. Pallor, edema, nutritional status, and lymphadenopathy were all examined and found to be unremarkable; however, the patient had jaundice. On examination, the abdomen was soft and nontender with no organomegaly and no signs of peritonism. Laboratory investigations, including liver function tests and complete blood count, were mildly elevated. Tumor marker carbohydrate antigen 19-9 was 12.3 U/mL (reference range: 0-37 U/mL) (Table 1).

**Table 1:** Laboratory investigations This table presents the patient’s key blood test results at admission, including CBC, LFTs, and inflammatory markers. Reference ranges are provided for comparison. Mild hyperbilirubinemia was observed with otherwise unremarkable LFTs, and the white blood cell count was within normal limits despite a history of fever CBC: complete blood count; LFTs: liver function tests; AST: aspartate transaminase; ALT: alanine transaminase; CRP: C-reactive protein; SGOT: serum glutamic oxaloacetic transaminase; SGPT: serum glutamic pyruvic transaminase

Parameter	Result	Reference range
Total leukocyte count	11,200/µL	4,000-11,000/µL
Hemoglobin	12.6 g/dL	13-17 g/dL (men)
Platelet count	260,000/µL	150,000-400,000/µL
Total bilirubin	2.3 mg/dL	0.2-1.2 mg/dL
Direct bilirubin	1.8 mg/dL	0.0-0.3 mg/dL
AST (SGOT)	38 U/L	10-40 U/L
ALT (SGPT)	42 U/L	10-45 U/L
Alkaline phosphatase	112 U/L	40-129 U/L
CRP	24 mg/L	<5 mg/L

According to the Tokyo Guidelines 2018, this case is best classified as Grade I (mild) acute cholangitis, characterized by local inflammation without systemic organ dysfunction. The patient fulfilled diagnostic criteria, including fever, elevated inflammatory markers (C-reactive protein), hyperbilirubinemia, and imaging suggestive of biliary obstruction, without evidence of hypotension, altered mental status, or organ failure.

A chest X-ray was performed, and it was normal. The patient also did not report any respiratory symptoms such as cough, hemoptysis, or shortness of breath. These findings further supported the diagnosis of isolated primary hepatic TB in the absence of pulmonary or miliary involvement.

A contrast-enhanced computed tomography (CECT) scan of the abdomen revealed hepatolithiasis accompanied by mild dilatation of the intrahepatic biliary radicles, primarily in the left lobe. Additionally, there was atrophy of the left hepatic lobe, with no evidence of a retained foreign body and a stricture noted at the origin of the left hepatic duct. Pneumobilia was also observed. The imaging features, in the background of prior biliary surgery, raised suspicion for cholangiocarcinoma or secondary biliary cirrhosis. There were no imaging features suggestive of intestinal TB.

**Figure 1:**
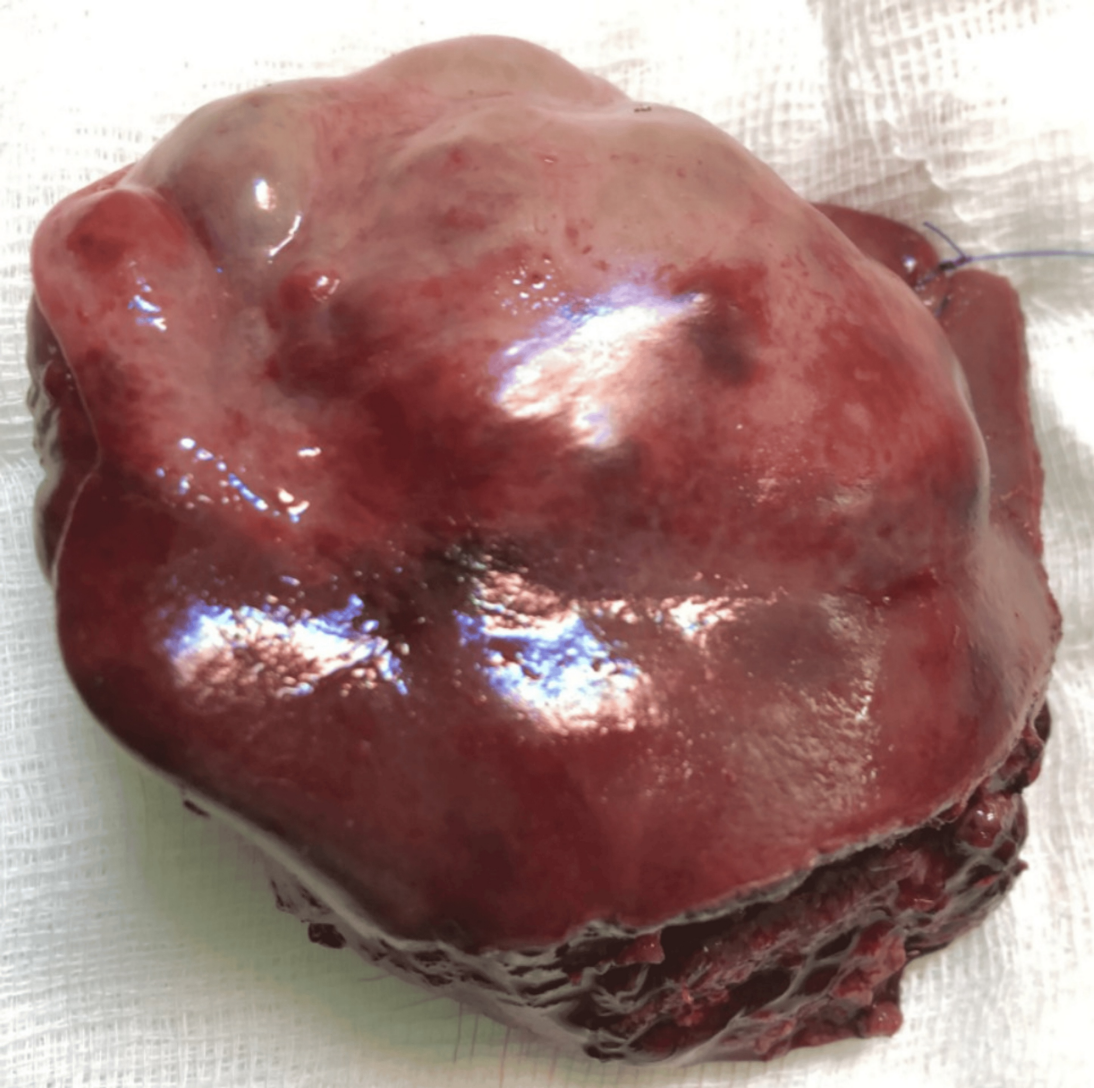
Gross specimen of left lateral segment of the liver The resected left lateral segment of the liver is shown. The lesion appears as a gray-white solid nodular mass without visible caseation, abscess, or necrosis. This segment was resected due to suspicion of malignancy. Precise measurement of the lesion was limited at the time of imaging due to the absence of an in-frame measuring scale. Final dimensions were confirmed via histopathological analysis

In view of the clinical and radiological findings, the patient underwent a left lateral segmentectomy of the liver. Intraoperatively, dense adhesion of the terminal ileum was found to involve the abdominal wall at the previous surgical scar site, and previous scar tissue necessitated limited ileal resection and primary anastomosis. There was no evidence of dense adhesions at the hepatoduodenal ligament or porta hepatis region. Intraoperatively, the liver appeared nodular and coarsely granular, and nodularity was confined to the left lobe of the liver, which appeared bulky and irregular. The right lobe was grossly normal in appearance. In view of the above findings, a left lateral segmentectomy was performed, and the gross specimen showed surface irregularity (Figure 1).

On gross examination, the histopathology of the liver specimen showed that the cut surface of the liver specimen revealed a firm, gray-white nodular lesion, without obvious areas of necrosis. The appearance was suggestive of a neoplastic lesion, and a hepatic tumor was the leading clinical differential diagnosis preoperatively. However, microscopic examination revealed nonnecrotizing granulomatous inflammation, which was consistent with tuberculous hepatitis, despite the absence of classic caseous necrosis. No malignant features were identified. Acid-fast bacilli staining was negative, and TB polymerase chain reaction (PCR) testing could not be performed as the possibility of hepatic TB was not clinically suspected preoperatively; moreover, imaging and clinical findings were suggestive of a neoplastic hepatic lesion. Therefore, TB-PCR was not requested on the resected specimen at the time of initial workup or intraoperatively. Given the histological features and endemic setting, empirical antitubercular therapy (isoniazid, rifampicin, ethambutol, and pyrazinamide) was started. The patient responded well clinically, further supporting the diagnosis. The patient has been followed up for six months postoperatively. During this period, he remained asymptomatic, showed good compliance with antitubercular therapy, and had no clinical or radiological signs of recurrence or residual disease.

## Discussion

Primary hepatic TB is defined as hepatic involvement by *Mycobacterium tuberculosis* in the absence of pulmonary or miliary TB. Hepatic TB accounts for less than 1% of all TB cases [1]. This condition remains extremely rare, even in TB-endemic regions, and often mimics malignancy in clinical presentation and imaging features [2]. Therefore, a high index of suspicion is required, particularly in endemic areas, to avoid unnecessary surgery and initiate timely antitubercular therapy. Key features that should prompt consideration of hepatic TB include prior history of TB or exposure, presence of systemic symptoms like low-grade fever or night sweats, atypical hepatic imaging findings (such as hypodense nodules or segmental atrophy without clear mass lesions), normal tumor markers despite concerning imaging, and histologic evidence of granulomatous inflammation. In cases like ours, where imaging mimics malignancy but systemic and biochemical features are not strongly suggestive, a preoperative biopsy may be prudent.

The pathogenesis of primary hepatic TB is not fully understood. It is hypothesized that tubercle bacilli reach the liver via the portal vein from a subclinical intestinal focus that later heals without a trace [5]. According to Levine [6], hepatic TB can be classified as follows: 1) primary liver TB, 2) TB cholangitis, 3) pulmonary TB with hepatic involvement, and 4) miliary TB with hepatic involvement.

Hepatic involvement in miliary TB occurs in 50-80% of cases, usually through hematogenous spread via the hepatic artery [6]. In contrast, primary hepatic TB is a rare entity [7,8]. Although the liver's low oxygen tension is unfavorable for mycobacterial growth [9], the organ’s rich blood supply and reticuloendothelial function facilitate granuloma formation. These granulomas, typically periportal, often cause minimal hepatic dysfunction and may remain asymptomatic.

Clinical features of hepatic TB are nonspecific. The most common signs and symptoms include fever, weight loss, abdominal pain, hepatomegaly, and elevated alkaline phosphatase levels [1]. Reported cases in the literature present with epigastric pain, anorexia, weight loss, or low-grade fever [2,7-10], though some are asymptomatic [2]. Our patient presented with cholangitis and had no history or signs suggestive of disseminated TB, reinforcing the diagnosis of primary hepatic TB.

Imaging features of hepatic TB are nonspecific and often resemble liver malignancies or metastases [2,6], making liver biopsy crucial when malignancy is suspected. In our case, hepatic TB was diagnosed histologically following surgery for biliary stricture with hepatolithiasis and atrophy of the left lobe of the liver. Histopathology in our case showed nonnecrotizing granulomatous inflammation. While caseating (necrotizing) granulomas are the classical hallmark of TB, nonnecrotizing granulomas have also been described in hepatic TB, particularly in early-stage or localized disease. Several case series have reported similar histological findings where acid-fast bacilli were absent, and diagnosis was made based on granulomatous inflammation in conjunction with clinical and radiological context [11]. Acid-fast bacillus (AFB) staining was negative, which aligns with literature reporting positivity in only about 40% of cases. TB-PCR, which has a sensitivity of 82% in detecting *Mycobacterium tuberculosis* in tissue samples, is considered the investigation of choice [11,12]. In this case, the possibility of hepatic TB was not clinically suspected preoperatively, as imaging and clinical findings were more suggestive of a neoplastic hepatic lesion. Therefore, TB-PCR was not requested on the resected specimen at the time of initial workup or intraoperatively. Mantoux and interferon-gamma release assays were not obtained at the time of presentation. While these tests cannot confirm hepatic TB specifically, they can provide supportive immunological evidence for latent or active TB infection, especially in cases where histopathology shows granulomatous inflammation but microbiological confirmation is lacking. Their inclusion in future diagnostic workups may aid in strengthening diagnostic confidence in similar presentations. Treatment consists of standard anti-TB treatment, tailored according to disease severity and liver function. This case report is limited by the absence of microbiological confirmation of TB, such as AFB staining, TB-PCR, or culture, which were not pursued due to a lack of clinical suspicion preoperatively. Imaging studies, including CECT and chest X-ray, were performed but are unavailable for reproduction. No colonoscopy or further gastrointestinal evaluation was done, as there were no suggestive symptoms. Additionally, histopathology images could not be accessed due to institutional restrictions. Despite these limitations, the diagnosis was supported by histological findings, the endemic context, and a favorable clinical response to antitubercular therapy.

## Conclusions

Primary hepatic TB is a rare and often overlooked diagnosis that can closely mimic hepatobiliary malignancies on clinical and radiological grounds. In TB-endemic regions, it is important to consider hepatic TB as a differential diagnosis for atypical liver lesions, particularly in patients with prior biliary surgery or nonspecific systemic symptoms. In this case, the diagnosis was made incidentally through postoperative histopathology, without classical microbiological confirmation or definitive imaging features. Although preoperative biopsy and further TB workup were not performed, the patient demonstrated clinical improvement with antitubercular therapy. This case underscores the diagnostic complexity of hepatic TB in atypical and incidental presentations. It highlights the importance of considering TB in the differential diagnosis of liver lesions in endemic regions, even when classical features are absent, and maintaining a high index of suspicion to avoid unnecessary major surgical interventions in similar scenarios.
